# Highly-scaled and fully-integrated 3-dimensional ferroelectric transistor array for hardware implementation of neural networks

**DOI:** 10.1038/s41467-023-36270-0

**Published:** 2023-01-31

**Authors:** Ik-Jyae Kim, Min-Kyu Kim, Jang-Sik Lee

**Affiliations:** Department of Materials Science and Engineering, Pohang University of Science and Technology (POSTECH), Pohang, 37673 Republic of Korea

**Keywords:** Electrical and electronic engineering, Information storage

## Abstract

Hardware-based neural networks (NNs) can provide a significant breakthrough in artificial intelligence applications due to their ability to extract features from unstructured data and learn from them. However, realizing complex NN models remains challenging because different tasks, such as feature extraction and classification, should be performed at different memory elements and arrays. This further increases the required number of memory arrays and chip size. Here, we propose a three-dimensional ferroelectric NAND (3D FeNAND) array for the area-efficient hardware implementation of NNs. Vector-matrix multiplication is successfully demonstrated using the integrated 3D FeNAND arrays, and excellent pattern classification is achieved. By allocating each array of vertical layers in 3D FeNAND as the hidden layer of NN, each layer can be used to perform different tasks, and the classification of color-mixed patterns is achieved. This work provides a practical strategy to realize high-performance and highly efficient NN systems by stacking computation components vertically.

## Abstract (web summary)

Hardware-based neural networks can provide a significant breakthrough in artificial intelligence. Here, the authors demonstrate an integrated 3-dimensional ferroelectric array with a layer-by-layer computation for area-efficient neural networks.

## Introduction

Neural networks (NNs) have made unprecedented improvements in intelligent tasks such as image and speech recognition^1,2^. However, with the current von Neumann-based hardware, the energy efficiency of NNs is limited by the data transfer process between the memory and processor units^2^. In-memory computing, in which computation is performed at the data storage, has been proposed to accelerate the speed of NN computation and address the von Neumann bottleneck^3,4^. Vector-matrix multiplication (VMM), which requires the multiplication of two numbers, is one of the main functions for the implementation of NN^5,6^. Previously, for the hardware implementation of VMM, a complex device structure with multiple adders was used, but after the emergence of artificial synapses, a new concept for VMM operation was proposed^1,3,7,8^. In artificial synapses, multiplication operations can be done by using Ohm’s law, which results in faster operation speed and lower energy consumption^5,6^. Also, the accumulation processes can be done by using Kirchhoff’s law^5^.

To implement the VMM operation, emerging two-terminal memories such as phase-change memory and resistive-switching memory have been investigated as artificial synapses^9–14^. Several NN models have been demonstrated using two-terminal memories. These emerging memory technologies successfully demonstrated neuromorphic characteristics and the NNs were implemented in a crossbar array structure, which has a potential for high-density arrays. However, additional access devices are required to reduce the leakage current in array structures to achieve accurate weight update and read processes^3,10,15,16^. As a memory cell can contain a single weight value to perform the designated tasks, additional memory elements or arrays are required when NNs are implemented in an array structure because different tasks such as feature extraction, error calculation, and classification should be done in different memory elements for parallel operations. Thus, the required number of memory arrays and chip size should be further increased for the implementation of complex NN models which contain multiple layers. One of the solutions for this issue can be the use of three-dimensional (3D) memory structures, which can stack the memory elements without increasing the area of the chip^17,18^. Alternatively, there are approaches using conventional memory devices such as NOR flash, NAND flash, and AND flash to implement NNs^19–22^. The flash memories based on the charge-trapping mechanism are one of the promising candidates for neuromorphic applications due to their high memory density and mature technology. However, as the neuromorphic applications require frequent updates of the synaptic weight (i.e., state) of the memory cells, NNs based on flash memories are only applicable for limited applications due to their high operation voltage and long latency^23,24^. Thus, further investigations for high-performance and 3D-compatible memory elements are required to develop hardware-based NNs.

The hafnia-based ferroelectric transistor is recently proposed as a promising candidate for next-generation memory devices including artificial synapses^15,24–36^. The hafnia-based ferroelectric transistor operates similarly to conventional charge-trap flash memory devices, where the threshold voltage (V_th_) can be tuned with applied gate voltages. In ferroelectric transistors, the V_th_ can be modulated by switching the polarization state of the ferroelectric layer, which can be done with faster speed and lower operation voltage compared to the charge-trap flash memory devices. The lower write voltage and faster operation speed of hafnia-based ferroelectric transistors than conventional charge-trap flash memory devices can be advantageous for neuromorphic applications^24,37^. By delicately controlling the polarization state of the ferroelectric layer, hafnia-based ferroelectric transistors show multilevel characteristics with high stability, which is favored in VMM operations^31,38,39^. Also, ferroelectric transistors have the potential to be adopted in high-density 3D NNs due to their high scalability and CMOS-compatibility. The high scalability of hafnia-based ferroelectrics can be advantageous for 3D memory applications^40,41^. Recent research demonstrated that hafnia-based ferroelectrics could be operated with a thickness under a few nanometers^42–44^.

In this work, we experimentally demonstrate an in-memory computable 3D ferroelectric NAND (FeNAND) array that utilizes a nanoscale vertical ferroelectric thin-film transistor (FeTFT) as a memory cell. We first propose a trench-based 3D array structure for FeTFTs, which has the potential to realize high-density hardware-based NNs. VMM operation is successfully demonstrated using the fabricated 3D FeNAND. We also show that the fabricated 3D FeNAND network can perform the accurate classification of patterns with a size of 4 × 2 pixels. Based on the experimental results, we also demonstrate that the proposed 3D FeNAND network can classify hand-written digit images with a high accuracy of 93.8%. Finally, by assigning each layer of 3D FeNAND to classify red, green, and blue colors, we show that the 3D FeNAND can perform a perfect classification of color-mixed patterns. This work presents a practical strategy to realize high-performance neuromorphic hardware systems based on 3D FeNAND.

## Results

### Fabrication and characterization of 3D FeNAND

**Fig. 1:**
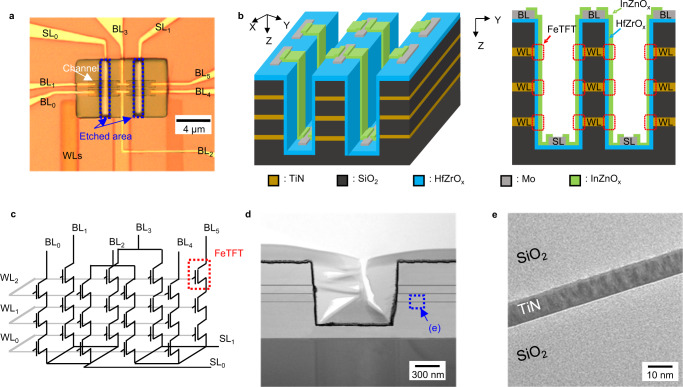
Demonstration of 3D ferroelectric NAND (FeNAND) using nanoscale vertical ferroelectric thin-film transistors (FeTFTs). **a** Optical image of the 3D FeNAND using nanoscale vertical FeTFTs. **b** Schematic illustration of 3D FeNAND (left) and cross-sectional view of 3D FeNAND with an effective channel area of 0.005 µm^2^. The thickness of TiN word-line (WL) and width of InZnO_x_ channel were 10 nm and 500 nm, respectively. **c** Equivalent circuit of the fabricated 3D FeNAND array. **d** Transmission electron microscope (TEM) image of the trench-based structure of 3D FeNAND array. **e** TEM image of SiO_2_/TiN/SiO_2_ WL stack.

The 3D FeNAND arrays with metal-ferroelectric-semiconductor-structured memory cells were fabricated using photolithography and the lift-off method (Supplementary Fig. 1)^24,41^. First, TiN word lines (WLs) and SiO_2_ layers were alternately deposited. Then, WL stacks were partially dry-etched to form trench-based structures. The HfZrO_x_ and Mo were used as ferroelectric gate insulating layer and source-/bit-line (SL/BL) electrodes, respectively. Oxide semiconductor InZnO_x_ layers were used as a channel (Fig. 1a). The fabricated 3D FeNAND had three layers, and eight memory cells were positioned at each layer (Fig. 1b, c). The device structure and thickness of each layer were confirmed using transmission electron microscopy (TEM). The thickness of the TiN gate electrode and the width of the InZnO_x_ channel were 10 nm and 500 nm, respectively, leading to an effective cell area of 0.005 μm^2^ (Fig. 1d, e). The thickness of the HfZrO_x_ and InZnO_x_ layers were 24 nm and 20 nm, respectively (Supplementary Fig. 2). Moreover, the crystal structure of the HfZrO_x_, which was deposited on the sidewall, was confirmed using TEM (Supplementary Fig. 3). The interatomic distance of HfZrO_x_ was about 0.294 nm, which corresponds to the interatomic distance of (111) orthorhombic phase of HfZrO_x_^45^. The trench-based vertical structure of the proposed 3D FeNAND can lead to higher memory density compared to gate-all-around (GAA) structures. When similar device dimensions are considered, the trench-based vertical structure can achieve double memory density compared to the GAA structure^46^. For example, in three WL stacks, three memory cells can be formed by a single etch hole in the GAA structure. For trench-based vertical structures, total six memory cells can be formed by a single etching. The electrical characteristics of 3D FeNAND memory cells were investigated under ambient conditions. The transfer characteristic of the 3D FeNAND memory cell located at the middle layer (WL1) was analyzed. By sweeping the WL voltage (V_WL_) between −6 and 6 V to the selected cell while applying a pass voltage (V_PASS_) of 2 V to the WLs of unselected cells, an n-type transfer characteristic with anticlockwise hysteresis was observed (Supplementary Fig. 4). This anticlockwise hysteresis is originated from the polarization switching of the ferroelectric HfZrO_x_ layer, and this property can be utilized for the program and erase operations in memory devices. Also, we considered the series resistance of the channel to project the maximum number of stacks. Based on the on- and off-state resistance of 3D FeNAND memory cells, more than ten times difference in string current is expected when 200 memory cells are vertically stacked in the proposed structure in the worst case. We believe much higher stacking will be possible by improving the channel mobility as well as the on-current characteristics of oxide semiconductors by optimizing the process parameters and/or adopting new channel materials. To avoid charge-trapping due to large sweep range of V_WL_, further measurements were done using voltage pulses, except the small read voltage for estimation of V_th_.

**Fig. 2:**
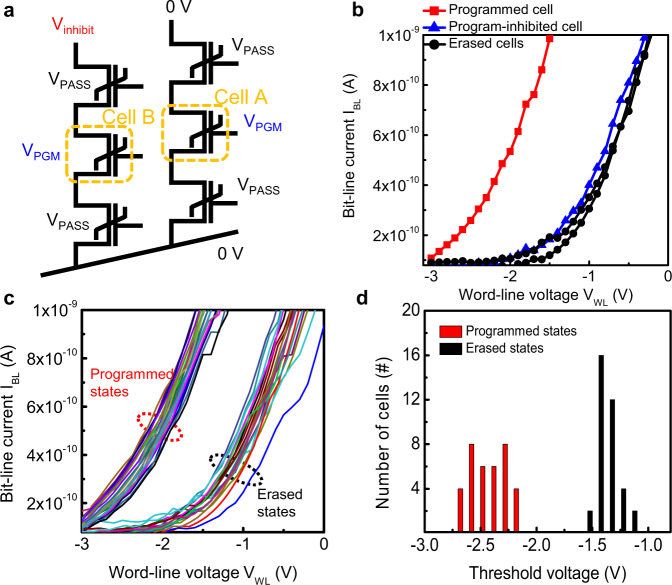
Operation characteristics of 3D FeNAND. **a** Equivalent circuits of 3D FeNAND and program operation. V_PGM_, V_PASS_, and V_inhibit_ stand for program, pass, and inhibit voltages, respectively. **b** I_BL_–V_WL_ curves of the selected memory cell and WL-sharing memory cell after erase and program operations. The program of the WL-sharing memory cell is prevented by the program-inhibit operation. The program-inhibit pulse with an amplitude of V_inhibit_ = 2.5 V is used for program-inhibit operation. **c** I_BL_–V_WL_ characteristics of memory cells in programmed and erased states. **d** V_th_ distribution of 24 memory cells in programmed and erased states.

In the proposed 3D FeNAND structure, unwanted programming may occur in memory cells that share the same WL with the selected memory cell during program operation. To prevent unwanted programming in unselected cells, a program-inhibit operation method was used (Fig. 2a). As an example, memory cells that shared the same WL were selected as programmed and program-inhibited cells, respectively. Before program operation, all memory cells in 3D FeNAND were erased by applying an erase pulse with an amplitude of −5 V and a width of 10 ms to the selected WL while 0 V was applied to the BLs and SL. Then, the selected memory cell was programmed by applying a program pulse with an amplitude of 4 V and a width of 10 ms to the selected WL. During the programming of the selected cell, the unwanted programming of the memory cell which shared the same WL was inhibited by applying program-inhibit pulses with an amplitude of 2.5 V and a width of 30 ms to BL. V_PASS_ of 2 V and a width of 30 ms was applied to unselected WLs. After erase and program operations, the states of memory cells were confirmed by sweeping V_WL_ from 0 V to −3 V. During these operations, programming of only the selected memory cell was achieved and the unwanted programming of the memory cell that shared the same WL was inhibited by program-inhibit operation (Fig. 2b). The proposed program-inhibit method was further analyzed using memory cells in each layer of the 3D FeNAND (Supplementary Fig. 5). First, all memory cells were erased by applying an erase pulse with an amplitude of −5 V and a width of 10 ms to the WLs, while 0 V was applied to the BLs and SL. Subsequently, the selected cells in WL0, WL1, and WL2 were sequentially programmed while the programming of other cells which shared the same WL was inhibited. After the erase and program operations, the states of memory cells were confirmed by sweeping V_WL_ from 0 V to −3 V. Using the program-inhibit method, the programming of the unselected cell was prevented. When a program-inhibit voltage with an amplitude of 2.5 V was applied to the BL, the channel potential of the nearest transistor (i.e., WL2) could be increased to 2.5 V. If the selected cell is in the WL2 layer, the program of the unselected cell that shares WL2 can be effectively prevented because the effective V_WL_ will be 1.5 V, which will not change the state of the 3D FeNAND memory cells. The program-inhibit efficiency can be decreased when lower cells should be inhibited because of the series resistance from the channel layers in highly stacked FeNANDs. The problem can be solved using diverse methods. First, the development of an optimized program-inhibit scheme can increase program-inhibit efficiency in highly stacked FeNANDs. For instance, increasing the program-inhibit voltage for cells positioned at lower WLs can be a viable solution. For ultra-high-density FeNANDs, select transistors (i.e., ground select line and string-select line) can be used for program-inhibit operations currently used in commercialized NAND flash memory devices, such as global and local self-boosted program-inhibit operations^47,48^. When the string-select line and ground select line are used, the program-inhibit can be achieved by applying program-inhibit voltage to BL, and turning off both select transistors. Once both select transistors are turned off, the channel is a floating node. At this point, when V_WL_ is increased, the potential of the channel will also be increased because of the capacitance coupling^47^. Owing to the small difference between the increased channel potential and V_WL_, the program-inhibit of the 3D FeNAND with select transistors can be achieved. Thus, for highly stacked 3D FeNANDs, select transistors can be used for program-inhibit operations.

The switching characteristics of 3D FeNAND memory cells were verified by applying voltage pulses with different amplitudes to the selected WL while applying a V_PASS_ = 2 V to unselected WLs. The V_th_ of the memory cell was changed with increasing pulse amplitudes (Supplementary Fig. 6). With an amplitude of 6 V, the device showed a clear V_th_ shift with a pulse width of 50 ns, and the device could be switched to a programmed state with a pulse width of 100 ns. To confirm the reliability of the 3D FeNAND, we investigated the data retention and endurance characteristics (Supplementary Fig. 7). For data retention characteristics, triangular program/erase pulses with an amplitude of ±4 V and pulse width of 10 μs were used for the program and erase operation, respectively. The states of the device were retained for 10^6 ^s at room temperature without failure. The endurance characteristics of the device were investigated by applying triangular program (4 V, 10 µs) and erase (-4 V, 10 µs) pulses. The V_th_ of the device was confirmed by sweeping V_WL_ from 0 to −3 V. The device showed stable switching characteristics for 10^6^ cycles. The device-to-device uniformity of 3D FeNAND memory cells was also characterized (Fig. 2c). The BL current (I_BL_) of the devices were measured after erase and program operations by sweeping V_WL_ from 0 V to −3 V. The erase and program operations were done by applying erase (−5 V, 10 ms) and program (5 V, 10 ms) pulses to the selected WLs, respectively. All devices showed a clear V_th_ shift to negative direction after program operation and the V_th_ at programmed and erased states were similar with a small distribution (Fig. 2d). The V_th_ shift to negative direction after applying positive V_WL_ indicated that ferroelectric polarization switching of the HfZrO_x_ layer affected the device properties^27,30,31^. In addition, device-to-device variations of 3D FeNAND memory cells were further investigated. To evaluate the device-to-device variation, I_BL_–V_WL_ curves at programmed and erased states were measured for 100 memory cells in 3D FeNAND. The memory cells showed similar I_BL_–V_WL_ characteristics, which confirmed the uniformity of the proposed 3D FeNAND (Supplementary Fig. 8). Furthermore, multilevel characteristics are required to realize neuromorphic properties in ferroelectric transistors. Using program pulses with different amplitudes, V_th_ tuning characteristics of the memory cell in 3D FeNAND were demonstrated (Supplementary Fig. 9). First, the memory cell was erased by applying an erase pulse (−5 V, 10 ms). After that, program pulses with different amplitudes of 3.5 V, 4 V, and 5 V were applied. As the amplitude of program pulses increased, I_BL_–V_WL_ curves shifted in a negative direction. The memory cell in 3D FeNAND showed four different V_th_ levels for ten cycles using program pulses with different amplitudes. As the effective cell area of the 3D FeNAND memory cell is 0.005 μm^2^, it is estimated that at least 50 grains are incorporated in the effective cell area of the device^49,50^. Thus, stable multilevel characteristics can be achieved due to the partial polarization characteristics of the HfZrO_x_ layer^51,52^. These results indicated that our devices have potential as memory devices with multilevel data storage capability. Because the trench-based 3D FeNAND structure can achieve higher memory density than GAA structures and the effective cell area of 3D FeNAND can be scaled down to 0.005 μm^2^_,_ the proposed 3D FeNAND can also be used as high-density memory devices. The experimental demonstrations of 3D FeNAND array operation containing program-inhibit operation, selective program, and multilevel data storage capability confirm the feasibility of 3D FeNAND for advanced memory applications.

### NN based on 3D FeNAND

**Fig. 3:**
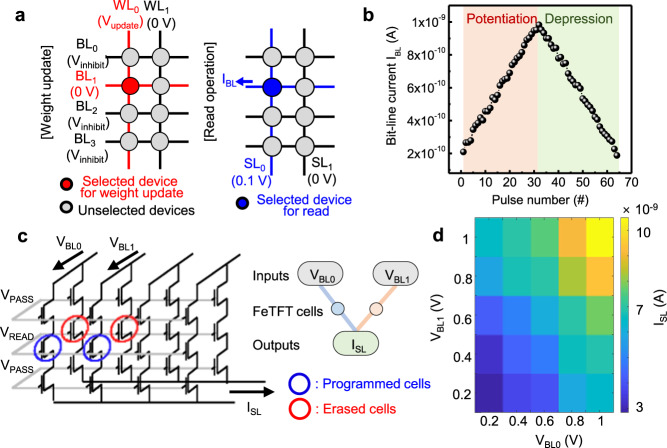
Demonstration of vector-matrix multiplication (VMM) using 3D FeNAND. **a** Weight update and read operation method for 3D FeNAND cell. For potentiation and depression operations, voltage pulses with incremental amplitudes were applied to the selected WL, and the selected BL was set to 0 V. Program-inhibit pulses with incremental amplitudes were applied to unselected BLs. The conductance of the devices was confirmed by measuring the current of the selected BL. **b** Potentiation and depression characteristics of 3D FeNAND cell. **c** Equivalent circuits (left) and schematic illustration (right) of VMM operation. Input voltages were applied to BLs and the product of VMM operation was measured at SLs. The output currents summed at the SLs were equal to the product of the input voltage applied to BLs and the conductance of memory cells. **d** Measured I_SL_ after VMM operation. BL voltages (V_BL0_ and V_BL1_) were used as the input vector and conductance values of ferroelectric memory cells were used as the weight matrix. The measured I_SL_ showed the summed output depending on the value of the input V_BL_.

For the implementation of NNs, the electrical characteristics of the artificial synapse (i.e., potentiation/depression) as well as the structural/operational characteristics of the array should be considered. The performance of the artificial synapse affects the accuracy of the NN, and the array should be able to perform VMM operation for the implementation of NNs. The device conductance of ferroelectric memory cells can be precisely tuned by controlling the partial polarization characteristics, which can be obtained by adjusting the amplitude of V_WL_. To identify the gradual conductance tunability of 3D FeNAND memory cells, potentiation and depression characteristics were investigated (Fig. 3a). For potentiation and depression, voltage pulses with incremental amplitudes and a width of 10 ms were applied to the selected WL, and the selected BLs were set to 0 V. The amplitudes of the potentiation and depression pulses increased from 2.5 to 3.74 V in a 40 mV step and from −3.5 to −4.74 V in a −40 mV step, respectively. Program-inhibit pulses with a width of 30 ms were applied to unselected BLs and SL. The amplitudes of the program-inhibit pulses for potentiation and depression operations were set to 2.0 V and −2.0 V, respectively. The conductance of the devices was confirmed by measuring the I_BL_ while a read voltage of 0.1 V was applied to the selected SL (Fig. 3b). The linearity of the potentiation and depression characteristics was evaluated using the following equations^27,31,53,54^,

1 $${{{{{{\rm{G}}}}}}}_{{{{{{\rm{pot}}}}}}}={{{{{\rm{B}}}}}}\left(1-{{{{{{\rm{e}}}}}}}^{\frac{-{{{{{\rm{P}}}}}}}{{{{{{{\rm{A}}}}}}}_{{{{{{\rm{pot}}}}}}}}}\right)+{{{{{{\rm{G}}}}}}}_{{{\min }}}$$

2 $${{{{{{\rm{G}}}}}}}_{{{{{{\rm{dep}}}}}}}=-{{{{{\rm{B}}}}}}\left(1-{{{{{{\rm{e}}}}}}}^{\frac{{{{{{\rm{P}}}}}}-{{{{{{\rm{P}}}}}}}_{{{\max }}}}{{{{{{{\rm{A}}}}}}}_{{{{{{\rm{dep}}}}}}}}}\right)+{{{{{{\rm{G}}}}}}}_{{{\max }}}$$

3 $${{{{{\rm{B}}}}}} \,=\, ({{{{{{\rm{G}}}}}}}_{{{\max }}}-{{{{{{\rm{G}}}}}}}_{{{\min }}})/\left(1-{{{{{{\rm{e}}}}}}}^{\frac{-{{{{{{\rm{P}}}}}}}_{{{\max }}}}{{{{{{{\rm{A}}}}}}}_{{{{{{\rm{pot}}}}}},{{{{{\rm{dep}}}}}}}}}\right)$$

where G_pot_ and G_dep_ are the conductance after potentiation and depression, respectively. P and P_max_ are the number of pulses and the maximum number of pulses, respectively. G_max_ and G_min_ are the maximum and minimum conductance, respectively^53,54^. In this equation, A_pot_ and A_dep_ represent the linearity of the potentiation and depression characteristics, respectively. By utilizing the equation, the 3D FeNAND memory cell showed high linearity of A_pot_ = 0.9842 and A_dep_ = 1.0125. Linear and symmetric potentiation and depression characteristics of the selected memory cell were achieved, which showed that the conductance of memory cells could be precisely tuned in highly scaled dimensions. In this work, the weights of memory cells were controlled using a pulse scheme with incremental pulse amplitudes. The use of an incremental pulse scheme can increase training time and power consumption due to the additional read process before weight updates. However, highly linear weight update characteristics can be achieved using incremental pulse schemes, which are required to achieve high recognition accuracy in neuromorphic applications^27^.

In in-memory computing technology, VMM is one of the most important functions required to implement a NN^5,6^. The VMM operation can be achieved in 3D FeNAND by simple methods. When input voltages are applied to each BL, the output current summed at the SL (I_SL_) is equal to the voltage multiplied by the conductance of each memory cell. Thus, in a 3D FeNAND array, each VMM operation uses the weights of the selected memory cells (Fig. 3c)^33,55^. The output I_SL_ is given by the product of the input V_BL_ matrix and conductance matrix,

4 $${{{{{{\rm{I}}}}}}}_{{{{{{\rm{SLj}}}}}}}=\mathop{\sum }\limits_{{{{{{\rm{i}}}}}}=1}^{{{{{{\rm{n}}}}}}}{{{{{{\rm{V}}}}}}}_{{{{{{\rm{BLi}}}}}}}{{{{{{\rm{W}}}}}}}_{{{{{{\rm{ij}}}}}}}$$

where W_ij_ is the weight of the ferroelectric memory cells connected to V_BLi_ and I_SLj_. We considered a VMM operation with four ferroelectric memory cells. An experimental demonstration of VMM operation was done using programming two memory cells and erasing other memory cells. V_BL_ from 0.2 V to 1 V with a step of 0.2 V was applied to the BLs while setting the cells of all other layers to the highly conductive state by applying V_PASS_ of 2 V to those WLs. The cell currents were collected at the SL. The measured output I_SL_ showed summed outputs depending on the value of input V_BL_, which showed the VMM operation capability of the suggested 3D FeNAND (Fig. 3d). The uniformity of VMM operation was also investigated (Supplementary Fig. 10). Four different 3D FeNANDs were used, and the output I_SL_ was measured under different I_BL_ values using the same method described above. The VMM outputs from different 3D FeNANDs were similar, which showed the reliability of VMM operation in nanoscale 3D FeNANDs. The reliability issues in ferroelectric transistors are originated from the degradation of the interfacial layer formed between HfZrO_x_ and the channel layer^24,28,56,57^. Utilization of oxide semiconductor channels can lead to an interfacial layer-free channel/HfZrO_x_ stack, which can improve the uniformity of the FeTFTs.

**Fig. 4:**
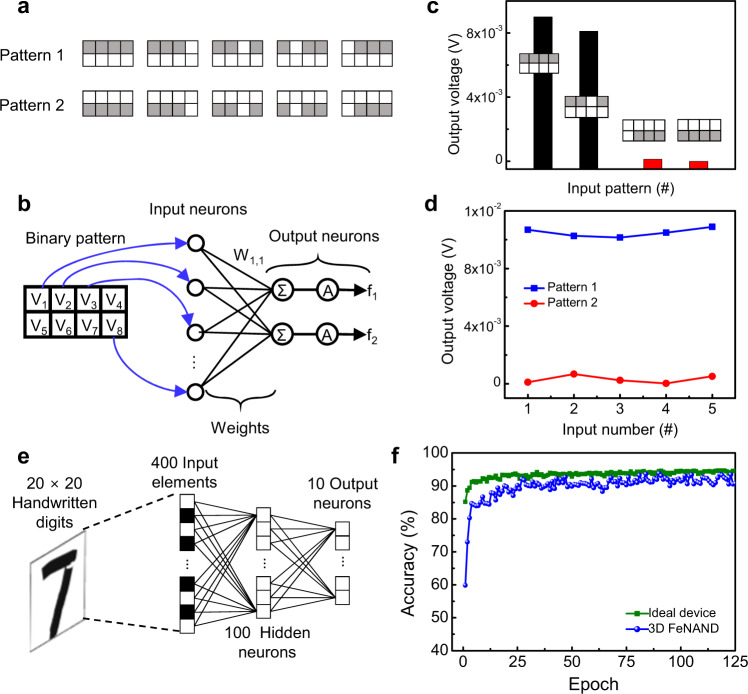
Image classification using 3D FeNAND array. **a** Example of training and test pattern set. Two-class image set which was comprised of a total of 20 training images of black and white patterns representing line patterns with a size of 4 × 2 pixels was used as a training and test image set. **b** Schematic illustration of binary image classification using 3D FeNAND. The value of each pixel in the input image was converted into voltages of 0 (black) or 1 V (white) and assigned to each BL in the 3D FeNAND array. The output I_SL_ was measured and used as the input for neurons (op-amp). **c** Neuron output voltages with different input patterns. The 3D FeNAND only showed a high output voltage when the black line was positioned over the white line. **d** Neuron output voltages according to repetitive input patterns. Pattern 1 (black line positioned over the white line) and pattern 2 (white line positioned above the black line) were used as input patterns. **e** Schematic illustration of MLP network for classification of MNIST hand-written digit images. 400 elements that corresponded to the number of pixels of input images (20 × 20) were used as the input and 100 hidden- and 10 output neurons were used for classification. **f** Comparison of simulated accuracies of MLP network based on 3D FeNAND and that based on ideal devices.

The multilayer perceptron (MLP) 3D FeNAND network was trained for the classification of a custom 2-class benchmark, which was comprised of a total of 20 training patterns with a size of 4 × 2-pixel. Black and white pixels were used, and black pixels in the same row represented a line (Fig. 4a, b)^58^. For the neuron output, operational amplifiers (op-amps) were connected to the SLs. The op-amps were used to convert the output current into the neuron output voltage (Supplementary Fig. 11)^59,60^. Two inverting op-amp circuits were used, and the first and second inverting circuits were utilized as the summation and activation layers, respectively. Before the training, the synaptic weights for pattern classification were calculated using the software-implemented network based on Python. Then, the calculated synaptic weights were imported to the weights of the 3D FeNAND memory cells^53,58,60–62^. The calculated synaptic weights were imported into the hardware by tuning the conductance of 3D FeNAND memory cells to the desired values using a write-and-verify method and the output current was measured at each string and summed. After training, the pattern classification was demonstrated. When input pattern 1, where black pixels were positioned at the top, was applied to the device the neuron output voltage of 8 × 10^−3^ V was observed (Fig. 4c). Moreover, when a single black pixel was flipped to a white pixel, a similar neuron output voltage was observed. However, with pattern 2, where black pixels were positioned at the bottom, the neuron output voltage was 1 × 10^−4^ V. The large difference in neuron output voltage under different patterns showed that the 3D FeNAND could classify the black and white pixels. Thus, it was shown that by using 3D FeNAND, the black and white pixels with different positions could be classified. Furthermore, the neuron output under repetitive inputs was investigated (Fig. 4d). For the same pattern, the neuron output was the same and clear differences were observed when different patterns were used as the input. Thus, the structural and operational feasibility of 3D FeNAND to perform classifications was confirmed using patterns with a pixel size of 4 × 2. Further optimization of the channel layer or use of oxide semiconductors with high mobility can result in stable VMM operations in highly stacked 3D FeNAND by decreasing the series resistance from channel layers.

Simulations based on the operation characteristics of 3D FeNAND were performed to confirm its performance when high-density 3D FeNAND is developed. The classification ability of the 3D FeNAND was further investigated using a Python-based simulation tool. An MLP network for the Modified National Institute of Standards and Technology (MNIST) dataset was simulated^62^. The MLP network was composed of 400 input elements, 100 hidden neurons, and 10 output neurons (Fig. 4e). The number of input elements corresponded to the size of the input MNIST images, which was 20 × 20 pixels. The synaptic characteristics of 3D FeNAND cells including the number of states, linearity, on/off ratio, and minimum/maximum conductance of potentiation/depression characteristics were implemented. Moreover, the device-to-device variation of potentiation/depression characteristics, which was measured for 20 memory cells in the 3D FeNAND array, was also considered in the simulations (Supplementary Fig. 12). In simulations, the MLP network based on a 3D FeNAND achieved an image recognition accuracy of 93.8%, which was comparable to the accuracy of 94% that the MLP network based on ideal synaptic devices achieved (Fig. 4f).

### Color-mixed pattern classification using 3D FeNAND

**Fig. 5:**
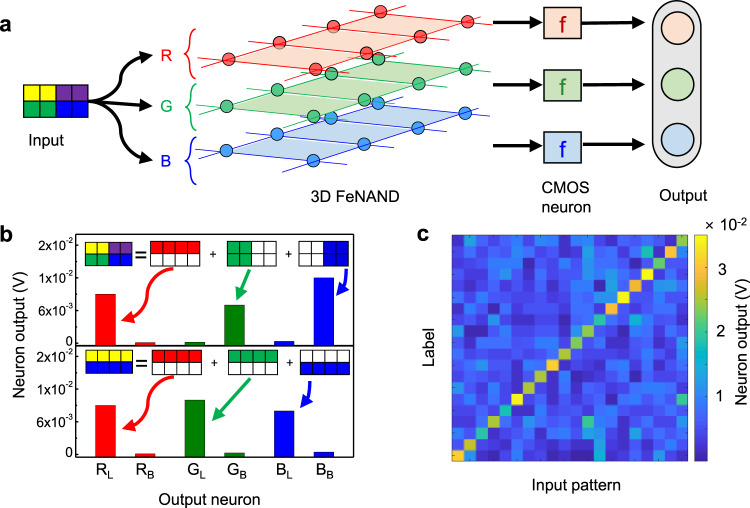
Color-mixed pattern classification using a 3D FeNAND-based neural network. **a** Schematic illustration of color classification using 3D FeNAND and CMOS neurons. The images fabricated by randomly adding the box and line patterns with red, green, or blue colors were used as test and training images. The patterns were passed through color filters (i.e., red, green, and blue), and the filtered value was converted as the input voltage to the BLs. The output I_SL_ was measured and used as the input for the neurons (op-amp). **b** Example of color-mixed pattern classification using 3D FeNAND-based neural network. The R_L_, R_B_, G_L_, G_B_, B_L_, and B_B_ stands for the red line, red box, green line, green box, blue line, and blue box, respectively. For the mixed pattern containing the red line, green box, and blue box, only the corresponding output neurons showed high neuron outputs. **c** Classification result for 20 input patterns. The summed neuron output showed high output only for the correct label.

A single layer of 3D FeNAND can classify binary patterns. Compared to two-dimensional (2D) arrays, 3D FeNAND can also process images with additional features such as color. With 2D arrays, an additional array is required to process additional features because each array is dedicated to specific tasks such as feature extraction and classification at the same time. Thus, it is hard to process images with extra features using a 2D array. Furthermore, recently developed software-based NN models require more than tens of billions of parameters, which will further increase the device area when 2D array is used to implement those models in neuromorphic hardware. However, 3D FeNAND can be stacked in a vertical direction with ultra-high density. The 3D FeNAND can be realized with a WL length of 10 nm and a trench-based structure, which can further increase the memory density. By utilizing all three layers of 3D FeNAND, color-mixed patterns can be successfully classified (Fig. 5a, b)^63^. We designated each FeNAND layer for the classification of red, green, and blue patterns. The test images with a pixel size of 4 × 2 were fabricated by randomly adding the box and line patterns with red, green, or blue colors (Supplementary Fig. 13). When 2 × 2 pixels and 1 × 4 pixels were designated to a specific color, the pattern was considered as a box and a line pattern, respectively. All cells were erased before training. For training, synaptic weights for pattern classification were calculated using the software-implemented network based on Python; subsequently, the calculated synaptic weights were imported to the weights of the 3D FeNAND memory. The patterns were passed through color filters (i.e., red, green, and blue filters), and the filtered values were used as the input voltage to the corresponding FeNAND layer. For example, when a color-mixed image consisting of the red line, green line, and blue box was applied to the 3D FeNAND, only output neurons corresponding to red line, green line, and blue box showed a high neuron output voltage (Fig. 5c). For 20 test images, the summed neuron output only exhibited a high output value (~0.03 V) at the correct label, which showed that the 3D FeNAND could be used for the classification of color-mixed images.

## Discussion

In this work, we have experimentally demonstrated a practical strategy to realize high-density, high-performance, and in-memory computable 3D FeNAND. First, we proposed a trench-based array structure for 3D FeNAND. The trench-based array structure is beneficial for higher memory density, as it can utilize both sidewalls as separate strings. We experimentally demonstrated that the WL length of 3D FeNAND could be scaled down to 10 nm which confirmed the high scalability of 3D FeNAND. In addition, the program-inhibit scheme of 3D FeNAND, which could also be utilized for memory applications, was experimentally demonstrated. Using the 3D FeNAND, diverse neuromorphic characteristics and in-memory computing features such as potentiation, depression, and VMM operations were demonstrated. The devices showed highly linear and symmetric potentiation/depression characteristics, and stable VMM operation characteristics. These stable operation characteristics of the 3D FeNAND are thought to be due to the utilization of oxide semiconductor channel materials, as it can prevent the growth of unwanted interfacial layers which can degrade the stability of the memory cells. Finally, we showed an experimental demonstration of color-mixed pattern recognition using 3D FeNAND. As the 3D FeNAND has a 3D structure, additional memory arrays or circuits are not required for different tasks. The computation can be done layer-by-layer, which can further decrease the chip size and increase area efficiency. By assigning each vertical layer in 3D FeNAND to classify different features (i.e., red, green, and blue colors), we showed that the classification of color-mixed patterns could be done. This work provides a practical strategy for hardware implementation of complex NNs using vertically stacked memory devices.

## Methods

### Materials

Hf[N(C_2_H_5_)CH_3_]_4_ [tetrakis(ethylmethylamido)hafnium (TEMAH)] and Zr[N(C_2_H_5_)CH_3_]_4_ [tetrakis(ethylmethylamido)zirconium (TEMAZ)] were purchased from UP Chemical, Korea. C_10_H_28_NSi_2_In_4_ (bis(trimethylsilyl)amidodiethyl indium, INCA-1) and Zn(C_2_H_5_)_2_ (diethylzinc, DEZ) were purchased from iChems, Korea. Si wafers with 300 nm-thick thermally grown SiO_2_ were used as substrates.

### Device fabrication

The devices were fabricated on a SiO_2_/Si substrate by photolithography, lift-off, and dry etching (Supplementary Fig. 1). Photolithography was performed using a mask aligner (400-LJ, Midas Systems) and i-line stepper (NSR 2205 i11D, Nikon). First, the SiO_2_/Si substrate was cleaned in acetone, ethanol, and deionized water for 15 min each. For SiO_2_/TiN/SiO_2_/TiN/SiO_2_/TiN/SiO_2_ stack, 10-nm-thick TiN WLs and 100-nm-thick SiO_2_ layers were sequentially deposited using DC sputtering and plasma-enhanced chemical vapor deposition (HiDep-SC, BMR Technology), respectively. Then SiO_2_/TiN/SiO_2_/TiN/SiO_2_/TiN/SiO_2_ layer was etched by dry etcher (Unity DRM, Tokyo Electron Ltd.) using sulfur hexafluoride (SF_6_) and Ar plasma. 24-nm-thick HfZrO_x_ layers were deposited by atomic layer deposition (ALD) using TEMAH, TEMAZ, and ozone at 280 °C. The 50-nm-thick Mo SL/BLs and 20-nm-thick InZnO_x_ channels were patterned using i-line stepper. The SL/BLs and channels were deposited by e-beam evaporation and ALD using INCA-1, DEZ, and ozone at 150 °C, respectively. Finally, the devices were annealed for 1 min at 500 °C under N_2_ gas to induce ferroelectricity in the HfZrO_x_ layer.

### Characterization

All the characteristics were measured under ambient conditions and at room temperature. The thicknesses of the HfZrO_x_ and InZnO_x_ were measured using atomic force microscopy (NX10, Park Systems). Optical images of the devices were captured using an optical microscope (LV100ND, Nikon). The cross-sectional images of the devices were obtained using a high-resolution transmission electron microscope (JEM-2200FS with image Cs corrector, JEOL). Before TEM observations, the samples were prepared using a focused ion beam (SII SMI3050SE, SII). The electrical characteristics were measured using a semiconductor parameter analyzer (4200A-SCS, Keithley Instruments) and a switching matrix (707B, Keithley Instruments). The polarization-voltage curves were measured using a pulse measurement unit (4225-PMU, Keithley Instruments). Sentaurus TCAD (Synopsys, Inc.) software was used for simulation. An MLP NN was measured using switching matrix and custom-built LabVIEW program. MNIST simulations were performed in Linux system with GCC, GNU make, CNU C libraries by using C++ code. The simulated MLP NN consisted of 400 input-, 100 hidden-, and 10 output neurons. The 400 input neurons corresponded to the 20 × 20 MNIST image, and the 10 output neurons corresponded to 10 classes of digits. The conductance ratio, linearity, and device-to-device variations of the 3D FeNAND memory cells were considered for simulations. For the simulation of NN based on ideal synapses, ideal synaptic properties including perfectly linear conductance modulation with a conductance ratio of 100, and 128 conductance states were used.

## Supplementary information

Supplementary Info

## Data Availability

All data that support the conclusions of this study are included in the article and the Supplementary Information file. These data are available from the corresponding author upon request.

## Code availability

The code used for simulation and array operation is available from the corresponding author with detailed explanations upon reasonable request.
