# Supplementary material for: Highly-scaled and fully-integrated 3-dimensional ferroelectric transistor array for hardware implementation of neural networks
Source: Nat Commun. 2023 Jan 31;14:504. doi: 10.1038/s41467-023-36270-0 (PMC9889761; doi:10.1038/s41467-023-36270-0)
Supplement: Supplementary file 1 — Supplementary Info [file 41467_2023_36270_MOESM1_ESM.pdf]

Supplementary Information

Highly-Scaled and Fully-Integrated 3-Dimensional Ferroelectric Transistor Array for Hardware Implementation of Neural Networks

Ik-Jyae Kim[1], Min-Kyu Kim[1], and Jang-Sik Lee[1]*

[1]Department of Materials Science and Engineering, Pohang University of Science and Technology (POSTECH), Pohang 37673, Republic of Korea

*E-mail: jangsik@postech.ac.kr

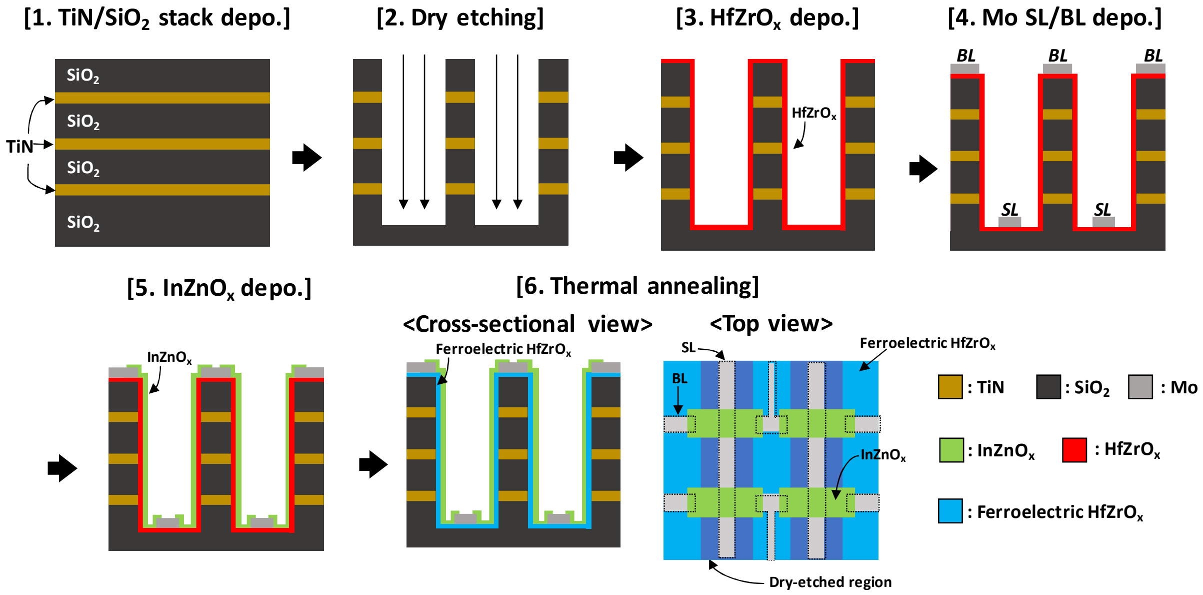

Supplementary Figure 1. Fabrication process of the 3D ferroelectric NAND (FeNAND). After the deposition of TiN WLs and SiO_2 layers, the etching process was done to form trench structures. Then, the $HfZrO_x$ gate insulator, Mo SL/BL, and $InZnO_x$ channel were deposited. Finally, annealing was done to induce ferroelectricity in the $HfZrO_x$ layer. 3D FeNAND is composed of three vertically stacked WLs.

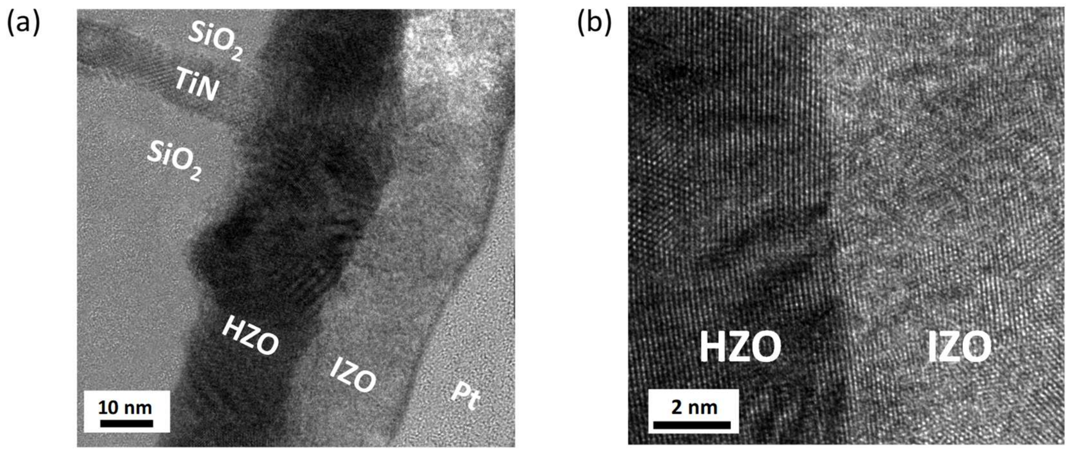

Supplementary Figure 2. Transmission electron microscope image of the (a) side-wall and (b) interface between $HfZrO_x$ and $InZnO_x$ of 3D FeNAND.

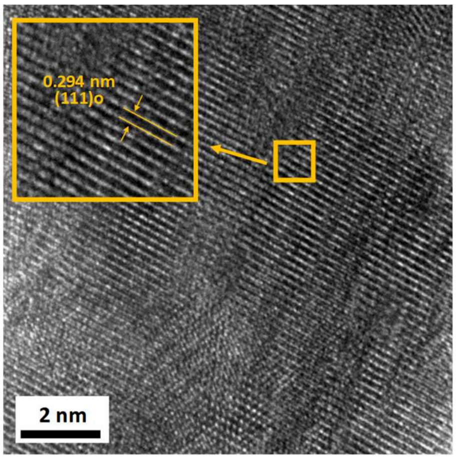

Supplementary Figure 3. Transmission electron microscope image of the $HfZrO_x$ layer in the sidewall of 3D FeNAND (inset: a magnified image of the corresponding area).

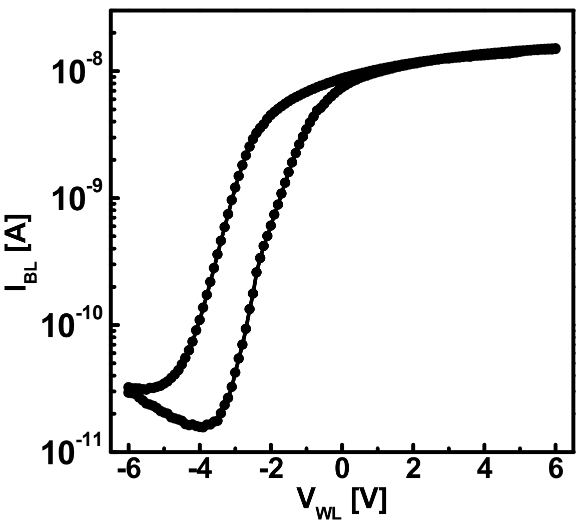

Supplementary Figure 4. Transfer characteristic of 3D FeNAND memory cell. The V_{PASS} of 2 V was applied to the unselected WLs.

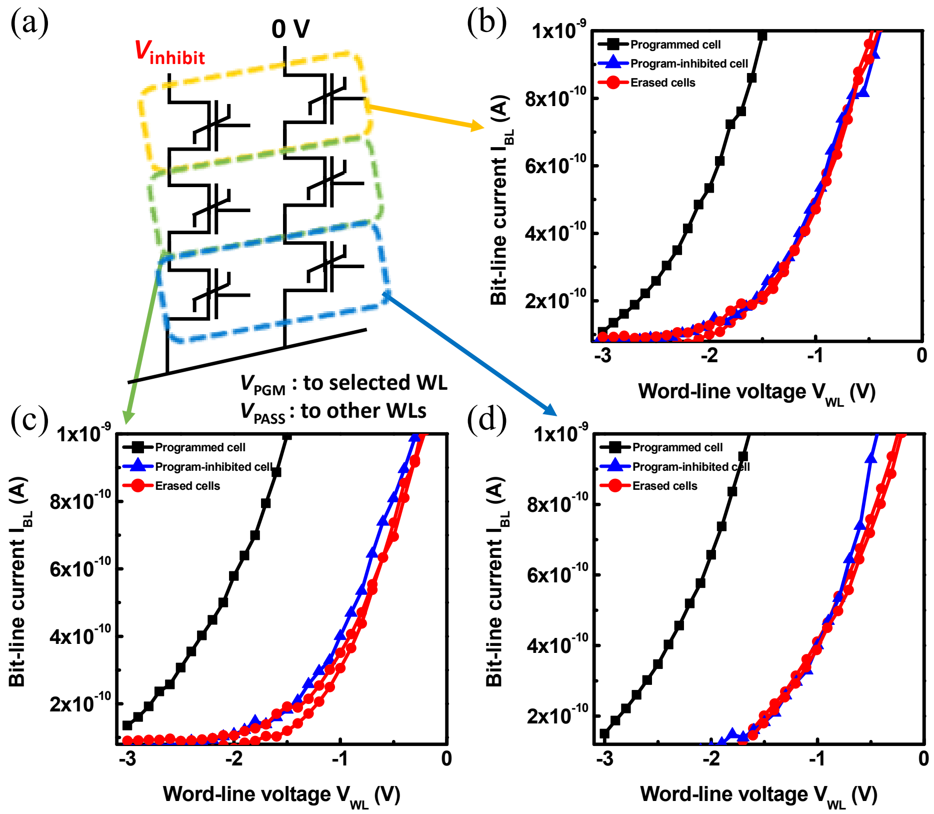

Supplementary Figure 5. Program-inhibit operation of 3D FeNAND. (a) Equivalent circuit of 3D FeNAND and program operation. V_{PGM}, V_{PASS}, and V_{inhibit} stand for program, pass, and inhibit voltage, respectively. I_{BL}-V_{WL} curves of the selected memory cell and WL-sharing memory cell after erase and program operation located at (b) top WL, (c) middle WL, and (d) bottom WL. The program of WL-sharing memory cell is prevented by program-inhibit operation.

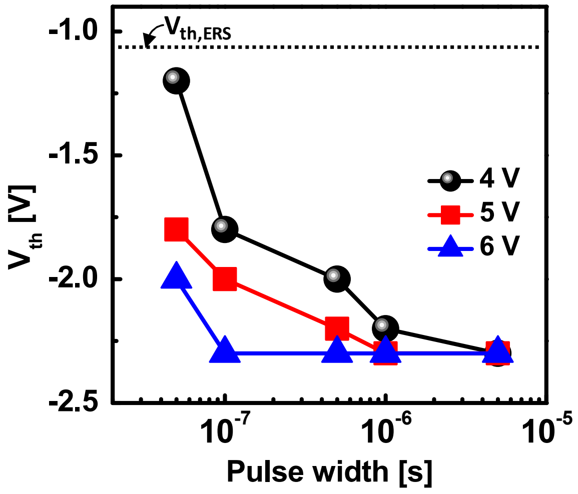

Supplementary Figure 6. Program speed of 3D FeNAND memory cell under different pulse amplitudes.

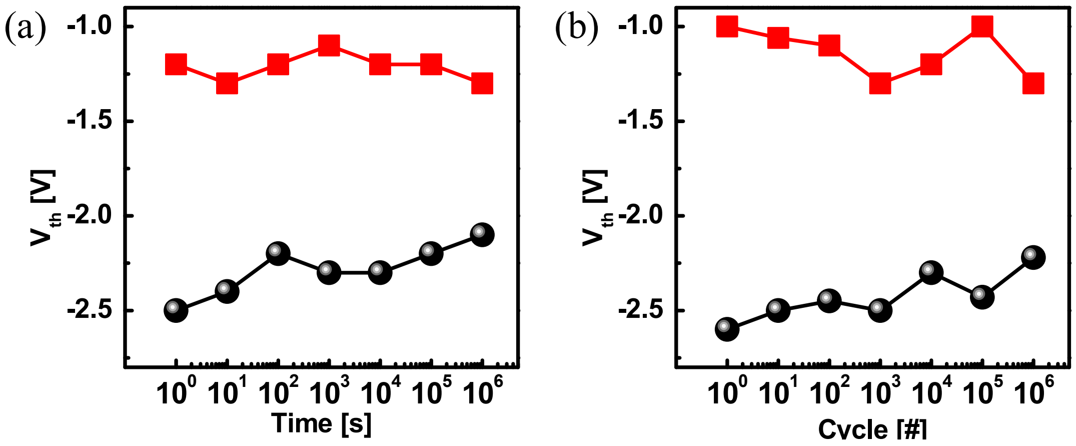

Supplementary Figure 7. (a) Data retention characteristics of 3D FeNAND memory cell for 10^6 s. The devices were programmed and erased using triangular voltage pulses of (4 V, 10 μs) and (-4 V, 10 μs), respectively. (b) Endurance characteristics of 3D FeNAND memory cell for 10^6 cycles. For endurance analysis, positive (4 V, 10 μs) and negative (-4 V, 10 μs) triangular pulses were used for program and erase operations, respectively.

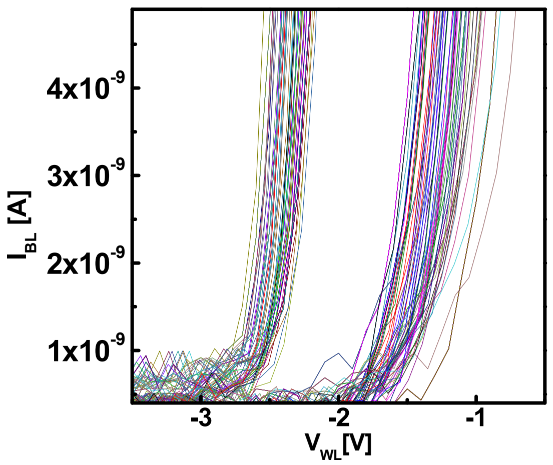

Supplementary Figure 8. I_{BL}- V_{WL} characteristics 100 3D FeNAND memory cells at programmed and erased states.

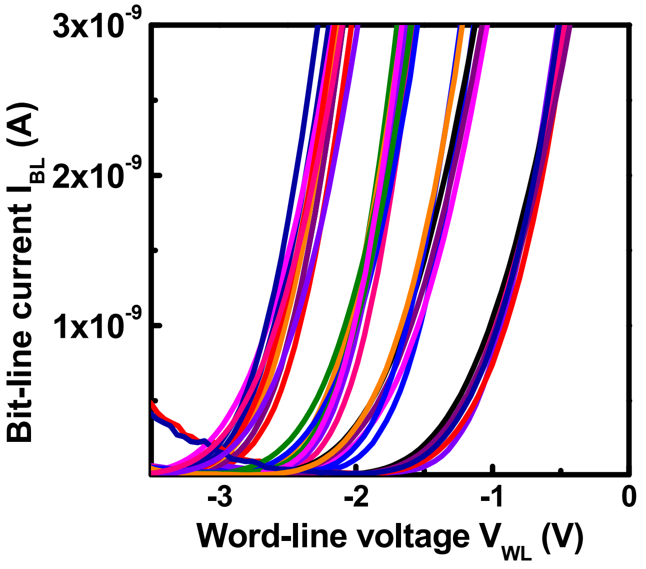

Supplementary Figure 9. V_{th} tuning characteristics of memory cell in 3D FeNAND.

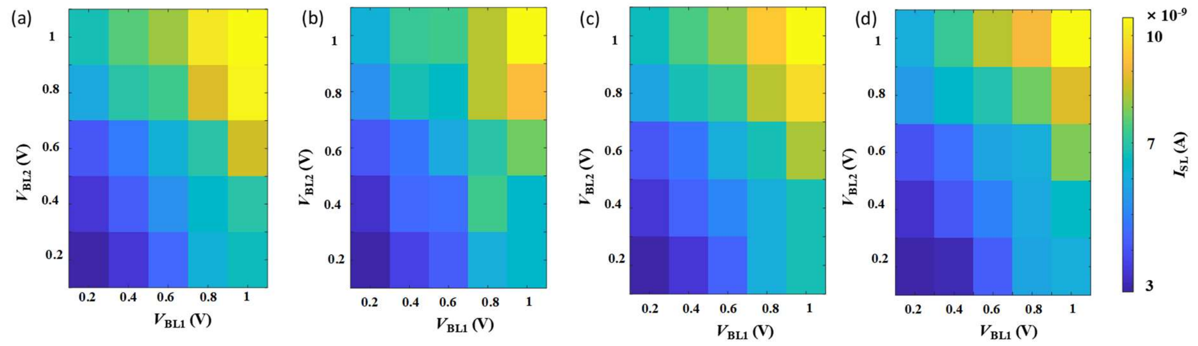

Supplementary Figure 10. (a-d) Device-to-device variation of VMM outputs. Four different 3D FeNAND array was used for VMM operations.

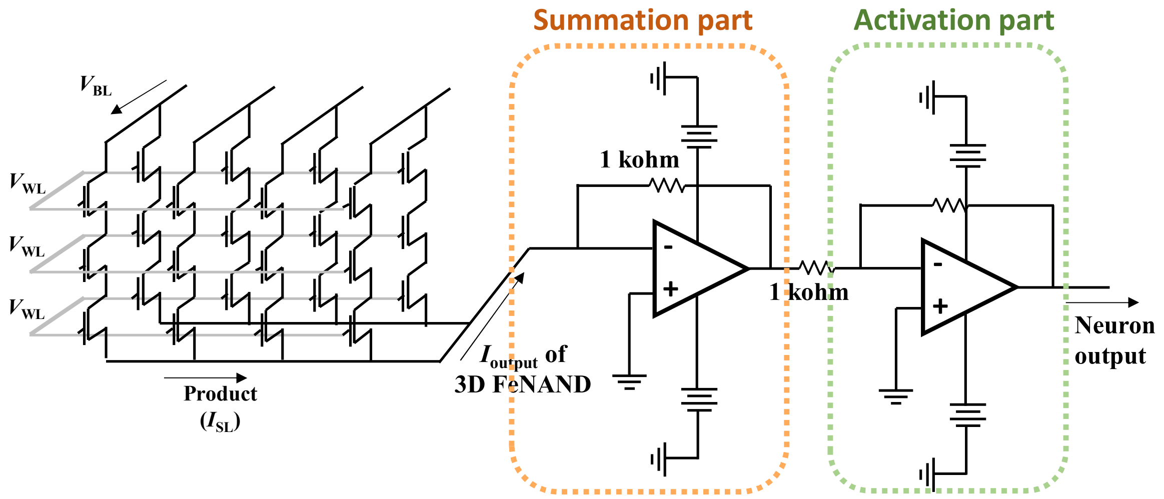

Supplementary Figure 11. Activation circuit composed of two op-amps for converting output I_{SL} to the neuron output voltage.

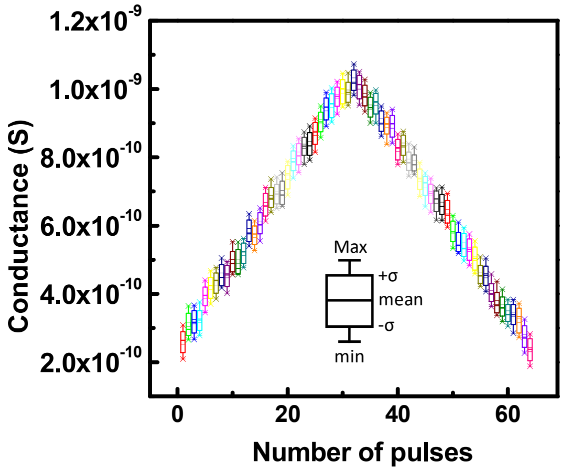

Supplementary Figure 12. Device-to-device variation characteristics of potentiation and depression cycles of 20 devices.

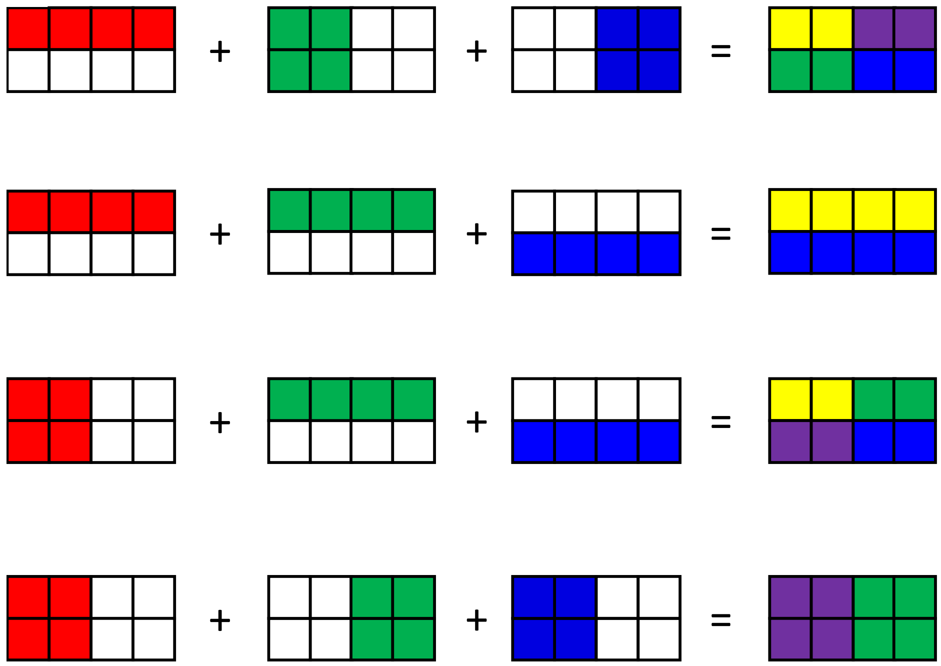

Supplementary Figure 13. Example of training/test patterns. Training and test patterns with a pixel size of 4×2 were fabricated by randomly adding the box and line patterns with red, green, or blue colors.
